# Diagnosis of Marfan Syndrome Following Progressive Myopia and Secondary Lens-Induced Angle Closure Crisis

**DOI:** 10.7759/cureus.62005

**Published:** 2024-06-09

**Authors:** Nicholas Fazio, Emily White, Andre Galenchik-Chan, Lauren Langman, Al Cossari, Robert Honkanen

**Affiliations:** 1 Ophthalmology, Renaissance School of Medicine, Stony Brook, USA; 2 Ophthalmology, Village Eye Care, Port Jefferson, USA; 3 Ophthalmology, Stony Brook Hospital, Stony Brook, USA

**Keywords:** acute angle closure, marfan syndrome, preventative care, genetic eye diseases, ectopia lentis

## Abstract

Marfan syndrome (MFS) is a well-described genetic connective tissue disease that heightens the risk of cardiovascular, ocular, pulmonary, and other emergencies in affected individuals. The wide range of phenotypic presentations, spanning from mild, chronic, and asymptomatic to acute and life-threatening, can pose challenges in diagnosing MFS when disease manifestations are subtle. We report a pathogenetic variant of MFS characterized by subtle systemic findings that was identified only after the patient presented with visual changes and pain associated with angle closure, despite a medical history indicating other pathologies linked to this condition. This case underscores the importance of recognizing the varied and sometimes subtle clinical features of MFS. Vigilance in identifying the constellation of findings associated with MFS can enhance its diagnosis and treatment outcomes by enabling appropriate and timely referrals for prophylactic evaluation and care to address potentially life-threatening complications.

## Introduction

Marfan syndrome (MFS) is an autosomal dominant genetic disorder that affects roughly 1 in 5000 individuals [1]. The disease affects the integrity of connective tissues and manifests in multiple tissues including the eye, heart, lungs, and skeletal system. It is most commonly due to mutations of the fibrillin-1 (FBN1) gene on chromosome 15. Numerous mutations and variants of this gene, nearly one thousand identified so far, contribute to the heterogeneous clinical presentation of MFS, with cardiovascular conditions being the most lethal [2,3]. Approximately 75% of individuals have an affected parent, but almost 25% of cases have a de novo pathogenetic variant of FBN1 [4]. Additionally, other patients can present with mutations of the TGFBR2 and TGFBR1 genes themselves, often referred to as 'Marfan syndrome type 2' [5,6].

The FBN1 encodes and regulates the protein fibrillin-1 which is a large extracellular matrix glycoprotein that serves as a structural component of microfibrils of the extracellular matrix and is expressed in various tissues including the eye, heart, lungs, vasculature, and skeletal system [1]. Within the eye, FBN1 is expressed in the iris, walls of Schlemm’s canal, cornea, and lens zonules. The lens zonules are the connective tissue fibers that attach the lens to the ciliary body and are responsible for lens centration and accommodation [7]. Missense mutations causing cysteine substitutions in the fibrillin-1 protein are associated with ectopia lentis, or displacement of the lens due to the loss of integrity of the lens zonules. The resulting displacement of the lens can cause angle closure and prevent the normal flow of aqueous humor from the anterior chamber, being one of the various causes of angle-closure glaucoma [8]. Ectopia lentis is the most common ocular manifestation in MFS due to an FBN1 mutation and is relatively specific to this disease [9]. A high rate (60%) of patients with MFS will develop lens dislocation in their lifetime [2]. In MFS, ectopia lentis is often asymmetrical, leading to amblyopia or anisometropia (a difference in visual acuity and refraction of the two eyes), which can be treated if diagnosed early [2].

Diagnosis of MFS can be challenging due to varied and often subtle clinical findings that can be missed even after examination by multiple providers and specialists. The diagnosis of MFS follows newer Ghent II criteria (2010) that primarily focus on the cardiovascular manifestation of aortic root dilatation and the ocular manifestation of ectopia lentis as cardinal features of the disease [10].

We present a case of MFS that was not diagnosed until an acute angle closure was precipitated by ectopia lentis; a condition where the natural crystalline lens of the eye is displaced anteriorly due to weak zonules. Diagnosis of MFS was not made until a comprehensive assessment of the patient revealed multi-systemic involvement. Genetic testing solidified the definitive diagnosis.

## Case presentation

A 56-year-old Caucasian male presented to an ophthalmologist following rapid vision loss and pain in his right eye. At the initial evaluation elsewhere, the patient was diagnosed with acute narrow angle-closure in the right eye and a laser peripheral iridotomy (LPI) was performed. The ocular pain subsided, and the intraocular pressure normalized, but the right eye blurriness persisted. He was advised to undergo cataract surgery. Because the patient was experiencing continued right eye blurry vision, he subsequently presented to this ophthalmology clinic for a second opinion on the diagnosis and best management.

At the presentation in this clinic, the patient’s complete medical history revealed mitral regurgitation, hypertension, hypothyroidism, and spontaneous pneumothorax. His family history was notable for a leaky cardiac valve in his father, although the patient specifically denied any family history of MFS. His body habitus was not particularly notable for his very tall stature or obvious arachnodactyly, although he did have pectus excavatum. The ocular exam showed visual acuity of 20/150 right eye (OD), and 20/20 left eye (OS) when wearing his moderate myopic spectacle correction. Vision improved to 20/25+ OD with a correction of -4.75-1.50 x 60, and to 20/20 OS with -4.00-0.75 x 15. This change represented about a 2-diopter myopic shift compared to his prior spectacle correction. Slit lamp examination was remarkable for a shallow anterior chamber in both eyes (OU). In the right eye, the anterior chamber shallowing was asymmetric and more prominent temporally. Anterior lens subluxation with minimal phacodonesis was noted OD. The intraocular pressure was normal at 16 mmHg in both eyes. The posterior segment exam including the optic nerve and retina was unremarkable except for asymmetric optic nerve cupping (cup disc ratio of 0.6 and 0.3 in the right and left eye, respectively). There was no retinal detachment. 

**Figure 1:**
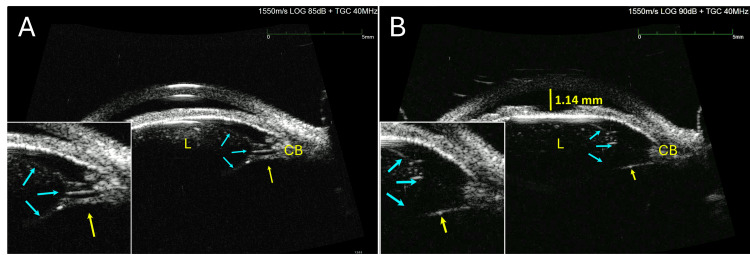
The UBM images of the anterior segment of the patient's right eye A: Intact lens zonules connecting the lens (L) to the ciliary body (CB).  The lens zonules (yellow arrows) attach both anteriorly and posteriorly to the lens equator. The edge of the lens is indicated by blue arrows. B: An area of abnormal zonule support is observed. Only one zonule fiber is identified on the side of the lens (yellow arrows), attached to the posterior aspect of the lens equator. The abnormal zonular support dislocates the lens anteriorly resulting in a shallow anterior chamber (1.14 mm) and angle closure. The edge of the lens is indicated by blue arrows. UBM: Ultrasound biomicroscope

After the gonioscopy showed an appositionally closed angle (no angle structures were visible), an ultrasound biomicroscope exam (UBM) was done to assess the lens and zonule status of the right eye. The UBM showed large areas of absent lens zonules nasally from 12:00 to 6:00 o’clock and confirmed anterior subluxation of the lens (Figure 1). The lens appeared normal in shape (did not appear microspherophakic). The central chamber depth of the right eye was only 1.14 mm compared to the left of 2.31 mm. The UBM of the left eye noted areas of zonular abnormality without complete absence.

Due to the high clinical suspicion for MFS based on the past medical history (mitral valve regurgitation and pneumothorax), family history of a leaky cardiac valve, and physical findings including pectus excavatum and apparent lens subluxation with secondary angle closure, the patient was recommended to undergo genetic testing. Genetic testing confirmed a pathogenetic variant of MFS, and the patient returned for appropriate follow-up care with his cardiologist for management and a genetics consultation to discuss familial risk. The patient’s ocular condition has remained stable. However, he has been lost to follow-up in our clinic. The patient has subsequently required cardiac thoracic surgery for Marfan-related complications, and his children have been diagnosed with MFS as well. 

## Discussion

**Figure 2:**
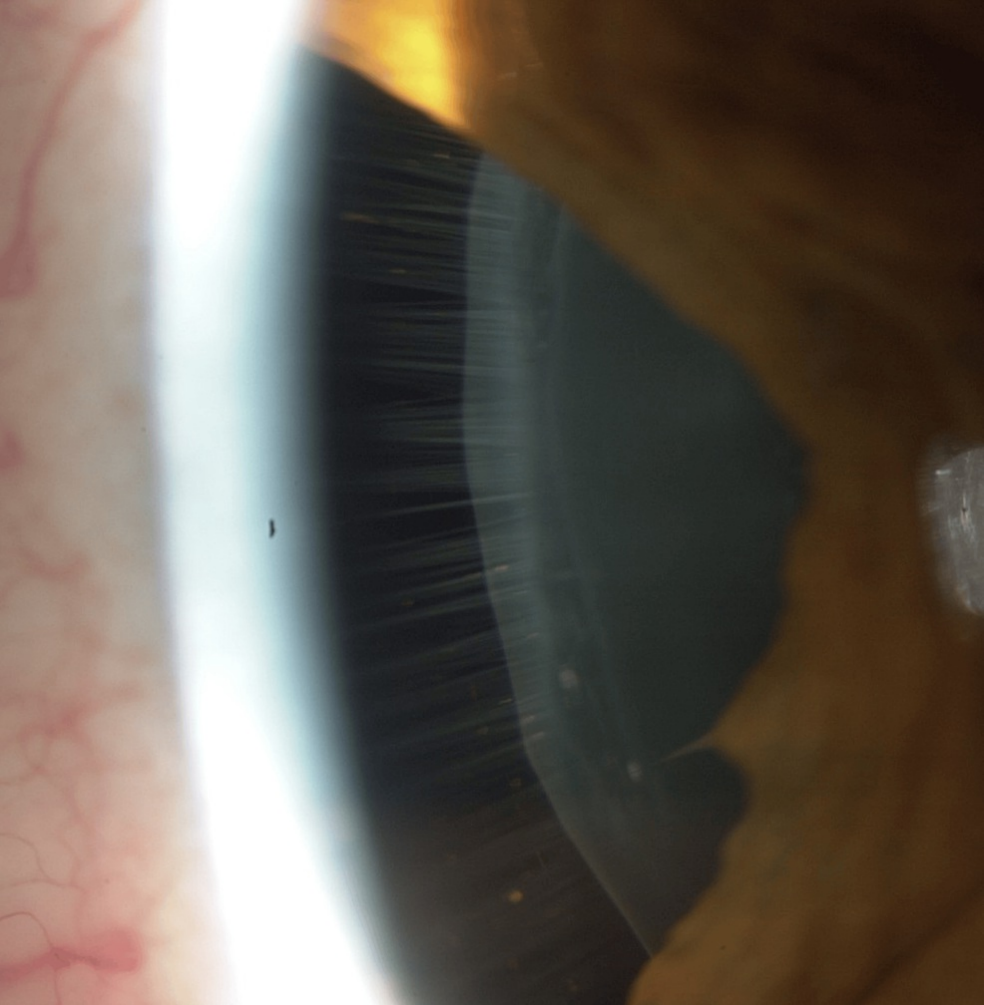
Slit-lamp photo of intact lens zonules seen through a peripheral iridectomy (unrelated case) These fibers maintain the position of the lens within the eye and are normally present around the entire circumference of the lens equator. Defects in the FBN1 gene can result in abnormal or absent zonules. Image credit: Author RH

Marfan syndrome is a serious medical condition many healthcare providers will see during their careers. This case highlights the importance for all healthcare providers to be vigilant and correctly identify those at risk for MFS to prevent the life-threatening complications associated with this condition. As demonstrated by this case, the wide array of clinical phenotypes in MFS, involving multiple organ systems, can render recognition of the condition based solely on clinical manifestations very challenging, potentially leading to the oversight of diagnosis in more subtle presentations [10]. The lens zonules, small connective tissue fibers, that attach the lens to the ciliary body (Figure 2) may lose integrity or become absent in MFS and may be the first presenting sign of MFS as ectopia lentis to clinicians. Notably, retrospective studies of patient populations with ectopia lentis have shown that these patients are not always referred for appropriate primary and cardiac care follow-up, even though ophthalmologists are aware of the causes of ectopia lentis and associated cardiovascular risks [11].

Physical appearance is often the first sign raising suspicion of MFS. However, it is difficult to distinguish MFS from Marfanoid habitus, a constellation of physical findings that can resemble MFS, including long limbs, long arm span, crowded oral maxilla with occasional high arched palate, arachnodactyly, and hyperlaxity. In such cases, a physical exam looking for specific physical findings including pectus carinatum (indentation of the sternum), reduced elbow extension, hindfoot deformity (fallen arches), gothic palate, down slanting palpebral fissure, lens subluxation, myopia 3> diopters, and remarkably high stature in women may help differentiate MFS from Marfanoid habitus when present [12]. In this particular case, the patient reported some findings that could raise suspicion for MFS, like myopia >3 diopters, prior pneumothorax, and ectopia lentis. However, his clinical appearance was otherwise so unremarkable that it did not raise suspicion for MFS despite being seen previously by multiple healthcare providers, including a cardiologist and ophthalmologist. 

In cases like this, where ocular findings may suggest MFS, a comprehensive family and medical history, revealing other systemic associations of MFS, can aid a provider in making the diagnosis. Considering the autosomal dominant inheritance pattern of MFS, a positive family history can help differentiate MFS from Marfanoid habitus, which lacks a genetic predisposition. Our patient self-reported a history of systemic conditions, raising suspicion for MFS, including a spontaneous pneumothorax. Although a spontaneous pneumothorax has been reported with MFS, this pertinent medical history had not previously been linked to MFS by any of his prior medical care providers. Furthermore, his vague family history of a leaky cardiac valve in a parent supported the possibility of MFS. However, the patient specifically denied being informed of an MFS diagnosis or any family members having received such a diagnosis.

Specific diagnosis of MFS follows Ghent II nosology, which was revised in 2010 to decrease the risk of premature or missed diagnoses [10]. Ghent II allows for diagnosis of MFS based on criteria including family history, aortic root dilation score, ectopia lentis, systemic score, and FBN1 mutation (Table 1). Compared to the original nosology, this new paradigm places more emphasis on cardiovascular and ocular manifestations of MFS, with aortic root aneurysm and ectopia lentis being cardinal features. However, even using this new diagnostic paradigm, a firm clinical diagnosis can be difficult to make, and genetic testing is often required to establish a diagnosis, as in this patient's case.

**Table 1:** Ghent II criteria Reproduced from *The Revised Ghent Nosology for the Marfan Syndrome* by Loeys et al. [10] with permission from the BMJ Publishing Group Ltd. Ao: Aortic diameter indicated by Z-score or aortic root dissection; EL: Ectopia lentis; FBN1: fibrillin-1 mutation; Syst: Systemic score; Z: Z-score

In the absence of family history	Criteria
	Ao (Z≥2) AND EL aortic dissection, and = MFS
	Ao (Z≥2) AND FBN1 = MFS
	Ao (Z≥2) and Syst (≥7 pts) = MFS
	EL AND FBN1 with known Ao= MFS
In the presence of family history	EL AND FH of MFS (as defined above) = MFS
	Syst (≥7 pts) AND FH of MFS (as defined above) = MFS
	Ao (Z≥2 above 20 years old, ≥3 below 20 years ) + FH of MFS (as defined above) = MFS
Scoring of systemic features	Wrist AND thumb sign = 3 (wrist OR thumb sign = 1)
	Pectus carinatum deformity = 2 (pectus excavatum or chest asymmetry = 1)
	Hindfoot deformity = 2 (plain pes planus = 1)
	Pneumothorax = 2
	Dural ectasia = 2
	Protrusio acetabuli = 2
	Reduced upper segment/lower segment ratio AND increased arm/height AND no severe scoliosis = 1
	Scoliosis or thoracolumbar kyphosis = 1
	Reduced elbow extension = 1
	Facial features (3/5) = 1 (dolichocephaly, enophthalmos, downslanting palpebral fissures, malar hypoplasia, retrognathia)
	Skin striae = 1
	Myopia >3 diopters = 1
	Mitral valve prolapse (all types) = 1
	Maximum total: 20 points, score ≥7 indicates systemic involvement

**Figure 3:**
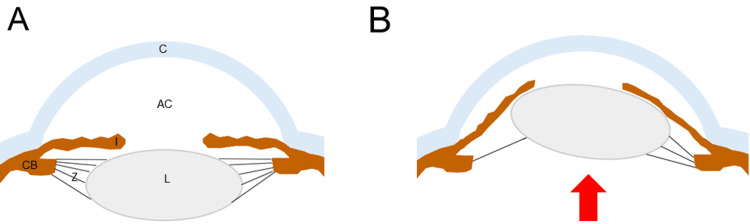
Graphic representation of ectopia lentis Panel A shows normal zonules (Z), which extend from the ciliary body (CB) to the lens (L), providing support and centration of the lens. Zonular support helps maintain a normal anterior chamber (AC) depth (distance from the cornea (C) to the lens). Panel B shows the marked absence of zonule support on the left side, which results in subluxation of the lens (red arrow) towards the cornea with resultant shallowing of the anterior chamber. Image credit: Authors RH and NF

The ocular presentation in this particular case is the most significant finding suggesting MFS. The patient’s history of long-standing chronic myopia with a recent progressive myopic shift at presentation is consistent with anterior subluxation of the lens due to MFS (which also induced the shallow anterior chamber). The acute secondary angle closure that the patient presented with occurs from anterior subluxation of the lens and a component of the pupillary block that prevents the normal egress of aqueous from the anterior chamber (Figure 3) and can be attributed to MFS [13]. Although this patient was successfully treated for acute secondary angle closure with laser iridotomy, he will need continued follow-up to ensure he does not develop glaucoma from another mechanism, as about 30% of patients with MFS develop primary open-angle glaucoma during their lifetime [2,14]. Additionally, among surveillance of patients with MFS, up to 10% will develop a retinal detachment, which may be secondary to the elongation of the globe due to decreased fibrillin-1 in the sclera [15]. 

This case highlights the need to take a broad approach encompassing family history, medical history, and specific attention to clinical findings and ancillary testing, including genetic analysis, to identify patients with MFS. The proper identification of these patients can prevent or minimize morbidity and mortality from associated systemic conditions.

## Conclusions

Marfan syndrome (MFS) is a complex autosomal dominant genetic disorder characterized by systemic manifestations affecting the eyes, heart, lungs, and skeletal system. The majority of cases result from mutations in the FBN1 gene. The diagnostic process can be challenging and necessitates vigilance by all healthcare providers for historical findings suggestive of the disease. The Ghent II criteria guide's specific MFS diagnosis emphasizes cardiovascular and ocular manifestations. Ocular findings, including ectopia lentis, are common and often highly suggestive of disease. The heterogeneity of clinical expression poses diagnostic challenges and underscores the importance for all healthcare providers to remain vigilant of this not infrequently seen condition. Early recognition through clinical and molecular investigations is crucial for improving life expectancy and implementing timely management.
